# Polymorphisms of DNA repair genes *XRCC1* and *XPD* and risk of primary open angle glaucoma (POAG)

**Published:** 2007-01-05

**Authors:** Mehmet Güven, Mustafa Ünal, Bahadir Batar, Ebru Eroğlu, Kazim Devranoğlu, Nevbahar Tamçelik, Didar Uçar, Ahmet Sarici

**Affiliations:** 1 Department of Medical Biology, Cerrahpasa Faculty of Medicine, University of Istanbul, Istanbul, Turkey; 2 Department of Ophthalmology, Akdeniz University Medical Faculty, Antalya, Turkey; 3 Department of Ophthalmology, Cerrahpasa Faculty of Medicine, University of Istanbul, Istanbul, Turkey

## Abstract

**Purpose:**

Oxidative DNA damage has been shown to have some role in the development of primary open angle glaucoma (POAG). In this study, we aimed to determine the frequency of polymorphisms in two DNA repair enzyme genes, Xeroderma pigmentosum complementation group D (*XPD*) codon 751 and X-ray cross-complementing group 1 (*XRCC1*) codon 399, in a sample of Turkish patients with POAG, and to evaluate their association with POAG development.

**Methods:**

We used polymerase chain reaction (PCR) and restriction fragment length polymorphism (RFLP), to analyze *XRCC1*-Arg399Gln and *XPD* -Lys751Gln polymorphisms in 144 patients with POAG and in 121 disease-free controls, who were of a similar age.

**Results:**

There was no significant difference in the genotype distribution between POAG patients and controls for each polymorphism (p>0.05). Allele frequencies were also not statistically different between the groups (p=0.46; OR: 0.77; 95% CI:0.42-1.43 for *XRCC1* 399Gln and p=0.88; OR: 0.92 95% CI: 0.50-1.67 for *XPD* 751Gln).

**Conclusions:**

Polymorphisms in *XPD* codon 751 and *XRCC1* codon 399 were not associated with risk of POAG in a sample of Turkish patients.

## Introduction

Glaucoma is an optic neuropathy characterized by a specific structural alteration of the head of the optic nerve accompanied by progressive damage to the visual field. Primary open-angle glaucoma (POAG) is the most common form of glaucoma, and it is one of the leading causes of irreversible blindness worldwide [1].

Although increased intraocular pressure (IOP) is a major risk factor for POAG, other concomitant factors affecting the eye play important roles including reactive oxygen species (ROS)-mediated oxidative damage. Oxidative DNA damage is significantly increased in the trabecular meshwork (TM) of glaucoma patients compared to controls, and the existence of a significant correlation between oxidative DNA damage and IOP in glaucoma patients has been reported [2-7]. Izzotti [8] reported that DNA damage may result in chronic degenerative diseases, including glaucoma, depending on the replication rate of the target cell population.

Recently, it has been hypothesized in many studies that polymorphisms in DNA repair genes reduce their capacity to repair DNA damage and thereby lead to a greater susceptibility to cancer or age-related diseases [9,10]. Although the exact pathogenetic mechanism of open angle glaucoma has not yet been fully clarified, the possible involvement of oxidative damage to DNA in POAG pathogenesis may indicate the role of DNA repair enzymes. Consistent with this hypothesis, Chen and Kadlubar [7] stated that polymorphisms in genes involved in antioxidant defenses and DNA damage repair may be genetic factors that predispose to an increased risk of glaucoma.

DNA repair enzymes continuously monitor chromosomes to correct damaged nucleotide residues generated by exposure to cytotoxic compounds or carcinogens. Repair of oxidative DNA damage is mediated by both base excision repair (BER) and nucleotide excision repair (NER) mechanisms [11,12]. Although hundreds of polymorphisms in DNA repair genes have been identified [13,14], their effects on repair function have not been well characterized. Among them, Xeroderma pigmentosum complementation group D (*XPD*), X-ray cross-complementing group 1 (*XRCC1*), and X-ray cross-complementing group 3 (*XRCC3*) have been frequently studied, and there is a growing body of evidence that polymorphisms of these genes may have some phenotypic significance [9,13].

XRCC1, a DNA repair protein involved in single-strand breaks (SSBs) and BER pathway, has been reported to be responsible for the efficient repair of DNA damage caused by active oxygen, ionization, and alkylating agents [15,16]. It is a multidomain protein that interacts with the nicked DNA and participates with at least three different enzymes, poly-ADP-ribose polymerase (PARP), DNA ligase III, and DNA polymerase β, to repair SSBs [17]. Three polymorphisms occurring at conserved sequences in the *XRCC1* gene were reported by Shen et al. [16]. These coding polymorphisms, resulting in amino acid substitutions, were detected at codons 194 (Arg-Trp), 280 (Arg-His), and 399 (Arg-Gln). In particular, the 399Gln polymorphism resulting from a guanine to adenine nucleotide substitution occurs in the PARP binding domain and may affect complex assembly or repair efficiency. Several studies have linked XRCC1-Arg399Gln polymorphism with biomarkers of DNA damage [18,19].

*XPD* encodes a helicase, which participates in both NER and basal transcription as part of the transcription factor IIH [9]. Mutations destroying enzymatic function of the XPD protein are manifested clinically in combinations of three severe syndromes, Cockayne syndrome, xeroderma pigmentosum, and trichotiodystrophy, depending on the location of the mutation [9]. Because XPD is important in multiple cellular tasks and rare *XPD* mutations result in genetic diseases, *XPD* polymorphisms may operate as genetic susceptibility factors. Premature aging has been reported in mice deficient in DNA repair and transcription because of a mutation in the *XPD* gene [20] Several single nucleotide polymorphisms (SNPs) in the *XPD* gene exons have been identified [9,17]. The *XPD*-Lys751Gln variant substantially modifies the amino-acid electronic configuration in a domain important for the interaction with helicase activator p44 and may produce the most relevant change in *XPD* function [21]. Lunn et al. [22] showed that individuals with the *XPD* codon 751 Lys/Lys genotype had a seven fold increased risk of suboptimal DNA repair.

Screening for the possible relationship between polymorphisms of DNA repair genes and POAG may contribute to understanding the pathogenesis of glaucoma and may be useful in the prevention of this disease. To our knowledge, no studies have examined the relationship between DNA repair enzymes polymorphisms and ocular disease susceptibility. As the polymorphisms in *XPD* codon 751 (Lys-Gln) and *XRCC1* codon 399 (Arg-Trp) are common in the population and have immediate functional significance, we determined the frequency of the polymorphisms in a sample of Turkish patients with POAG, and evaluated their association with POAG development.

## Methods

This case-control study included a total of 144 patients with POAG and 121 disease-free controls. The eligible patients with POAG and controls were selected consecutively at Istanbul University Cerrahpasa Medical Faculty Ophthalmology Department. The research followed the tenets of the Declaration of Helsinki, and all patients signed an informed consent form after they received an explanation of the nature of the study.

Each subject underwent a complete ophthalmological examination. Glaucoma subjects were defined by the presence of pathological cupping of the optic disc, and a glaucoma hemifield test (GHT) outside normal limits with reproducible visual field defects (VFD) at the same location on two consecutive visits, and an IOP higher than 21 mmHg. The number of patients with mild, moderate, and severe VFD was 52 (36%), 56 (39%) and 36 (25%), respectively. 106 (74%) of the patients had IOP levels between 21 and 28 mmHg. The rest of them (26%) had higher than 28 mm Hg.

Patients with a history of eye surgery before the diagnosis of glaucoma or with an evidence of secondary glaucoma, such as exfoliation, pigment dispersion, or uveitis were excluded. The mean age of the glaucoma group was 61.3±6.9 years (ranging from 48 to 79). Of these, 73 (51%) were at or lower than 60 years of age, and 70 (49%) were men.

Normal subjects presented with nonspecific ocular complaints, such as conjunctivitis, refractive disorders, blepharitis, etc., to our outpatient department. They were age-matched healthy volunteers with normal ocular examination including an IOP lower than 21 mmHg and GHT within normal limits. The mean age of the control group was 59.1±5.8 years (ranging from 51 to 75). Of these, 63 (53%) were at or lower than 60 years of age, and 66 (55%) were men.

### Blood samples and DNA isolation

Venous blood samples were obtained from patient and control groups and collected into EDTA tubes. Immediately after collection, whole blood was stored in aliquots at -20 °C until use. Genomic DNA was extracted from whole blood using a NucleoSpin DNA purification kit (Macherey-Nagel GmbH, Duren, Germany) according to the manufacturer's instructions.

### Genotyping of *XRCC1* codon 399

*XRCC1* genotypes were determined by polymerase chain reaction restriction fragment length polymorphism (PCR-RFLP). An Arg->Gln substitution in exon 10 (codon 399) was amplified to form an undigested fragment of 242 bp using primers described in reference [23]: 5'-CCC CAA GTA CAG CCA GGT C-3' (forward) and 5'-TGT CCC GCT CCT CTC AGT AG-3' (reverse). After an initial denaturation at 94 °C for 4 min, there were 35 cycles of 30 s at 94 °C, 30 s at 60 °C, and 30 s at 72 °C, and then a final extension step of 10 min at 72 °C. PCR products were digested with *Msp* I (Promega, Madison, WI) at 37 °C overnight and analyzed on 2% agarose gel. Arg/Arg individuals had 94 and 148 bp fragments, Arg/Gln individuals had 94, 148, and 242 bp fragments, and Gln/Gln individuals had only a 242 bp fragment.

### Genotyping of *XPD* codon 751

*XPD* genotypes were determined by PCR-RFLP. A Lys->Gln in exon 23 (codon 751) was amplified to form an undigested fragment of 436 bp using primers described in reference [24]: 5'-GCC CGC TCT GGA TTA TAC G-3' (forward) and 5'-CTA TCA TCT CCT GGC CCC C-3' (reverse). After an initial denaturation at 94 °C for 3 min, there were 38 cycles of 45 s at 94 °C, 45 s at 60 °C, and 60 s at 72 °C, and then a final extension step of 7 min at 72 °C. PCR products were digested with *Pst* I (Promega) at 37 °C overnight and analyzed on a 3% agarose gel. *Pst* I digestion resulted in two fragments of 290 and 146 bp for the wild-type homozygotes (Lys/Lys); three fragments of 227, 146, and 63 bp for the variant homozygotes (Gln/Gln); and four fragments at 290, 227, 146, and 63 bp for the heterozygotes (Lys/Gln).

### Statistical analysis

Ages of the patient and the control groups were compared with Student's t-test. Chi-square analysis (χ^2^ tests) was used to compare the gender distribution, test the association between the genotypes and alleles in relation to the cases and controls, and test for deviation of genotype distribution from Hardy-Weinberg equilibrium (HWE). A p<0.05 was used as the criterion of significance. The odds ratio (OR) and their 95% confidence intervals (CI) were calculated to estimate the strength of the association between polymorphism genotype alleles and patients and controls.

For the total sample size used in this study, we found an association with an OR 2.5 or more for acquiring a polymorphism could be detected with 80% or more power.

## Results

As shown in Table 1, the study included 144 cases with POAG and 121 healthy controls. The groups were not statistically different with respect to age (p=0.21) and gender (p=0.40).

**Table 1:** Demographic data of primary open angle glaucoma patients and disease-free controls used in this study.

	**Glaucoma group**	**Control group**	**p-value**
Number	144	121	
Gender			0.40
Male, n (%)	70 (49)	66 (55)	
Female, n (%)	74 (51)	55 (45)	
Age (years)			
Mean±SD	61.3±S6.9	59.1±S5.8	0.21
Range	48-79	51-75	

A total of 265 Turkish subjects were examined. No significant difference was observed in respect to gender or age between glaucoma (144 patients) and healthy controls (121 patients). Data for age is expressed as mean±standard deviation and range of the ages. Data for gender is number with percentages in parentheses.

The distributions of the *XPD*-Lys751Gln and *XRCC1*-Arg399Gln genotypes were in accordance with HWE among the controls (p=0.25, p=0.06, respectively) and the cases (p=0.20 and p=0.16, respectively). No statistically significant differences were observed in the alleles or in the genotype frequencies of the *XRCC1*-Arg399Gln and *XPD*-Lys751Gln gene polymorphisms between the control group and the patients with POAG (Table 2).

**Table 2:** Distribution of allele and genotype frequency of *XRCC1*-Arg399Gln and *XPD*-Lys751Gln polymorphisms in glaucoma patients and healthy controls.

**Gene**	**Controls n (%)**	**Patients n (%)**	**OR (95% CI)**	**p-value**
XPD				
Lys/Lys	25 (21)	33 (23)	Reference	
Lys/Gln	74 (61)	87 (60)	0.89 (0.46-1.70)	0.82
Gln/Gln	22 (18)	24 (17)	0.82 (0.35-1.93)	0.77
A (Lys) allele frequency	0.51	0.53		
C (Gln) allele frequency	0.49	0.47	1.08 (0.60-1.96)	0.88
XRCC1				
Arg/Arg	34 (28)	56 (40)	Reference	
Arg/Gln	76 (63)	78 (55)	0.62 (0.35-1.10)	0.11
Gln/Gln	11 (9)	10 (5)	0.55 (0.19-1.58)	0.33
G (Arg) allele frequency	0.6	0.65		
A (Gln) allele frequency	0.4	0.35	0.77 (0.41-1.43)	0.46

A two-side χ^2^ test was used to compare the distribution of the genotypes and alleles between cases and controls. Conditional logistic regression analysis was performed to calculate the odds ratios (OR) with 95% confidence intervals (CI) for estimating the strength of the association between polymorphism genotype alleles and patients and controls. No statistically significant differences were observed in the alleles or in the genotype frequencies of the *XRCC1*-Arg399Gln and *XPD*-Lys751Gln gene polymorphisms between the control group and the patients with POAG.

To explore whether or not a selective effect of polymorphisms exist in particular patient subgroups, we also analyzed the results by stratifying subjects depending on their age (less than or equal to 60 versus >60), IOP (between 21 mmHg and 28 mmHg, and higher than 28 mmHg), and visual field defects (mild, moderate, severe). Statistical analysis revealed no association between the alleles or the genotype frequencies of the *XRCC1*-Arg399Gln and *XPD*-Lys751Gln gene polymorphisms and the patient subtypes (>0.05).

## Discussion

Oxidative damage to DNA is the seemingly inevitable consequence of cellular metabolism. Elevated levels of oxidatively damaged DNA have been measured in numerous diseases including many types of cancer, neurologic disorders, coronary heart disease, hepatic diseases, and atopic dermatitis. As a result, it has been hypothesized that such damage plays an integral role in the etiology of these diseases [25].

ROS-mediated oxidative damage has been shown in the pathogenesis of POAG [2,4,5,8]. Oxidative stress also appears to be involved in the neuronal cell death affecting the optic nerve in POAG [2,3]. ROS can induce base damage, abasic sites, single strand breaks, and double-strand breaks in DNA [13]. Some studies have reported that oxidative DNA damage is significantly increased in the TM of glaucomatous patients compared to controls. Also, the existence of a significant correlation between oxidative DNA damage and IOP in glaucoma patients has been reported [2-7]. DNA damage in nonreplicating cells, such as in TM, may trigger apoptosis and death of cells that cannot be replaced, thus causing tissue degeneration [26]. Consistent with this, some studies showed decrease in TM cells with age and POAG [27,28].

It has been also reported that IOP increase and severity of visual-field defects in glaucoma patients parallel the amount of oxidative DNA damage affecting TM [2]. Sacca et al. [4] reported that oxidative DNA damage in the human TM may represent an important pathogenetic step in POAG because it could induce human TM degeneration, favoring an IOP increase, thus priming the glaucoma pathogenetic cascade. Izzotti et al. [5] found increased levels of 8-hydroxy-2'-deoxyguanosine (8-OH-dG) levels, an indicator of oxidative DNA damage, in glaucoma patients and showed an oxidative stress-dependent accumulation of DNA damage in the TM region. Interestingly, increased 8-OH-dG levels have been found previously to be related with *XRCC1* polymorphism [29].

POAG typically occurs after the age of 40 years, and its prevalence increases with age [5]. The recent hypothesis is that common variants SNPs in the population may contribute significantly to genetic risk for common diseases including age-related disorders. Functional variants of DNA replication and repair genes also might be expected to be highly significant to cancer and aging [10]. As an example, Duell et al. [30] showed that *XRCC1* Arg399Gln polymorphism caused more markers of DNA damage among older subjects than younger subjects.

It has been known for a long time that many primary eye diseases, including POAG, have genetic components. At least 15 genetic loci have been mapped for POAG [29]. Also, polymorphisms of the related genes, a subject of research in understanding the pathogenesis of POAG, have been shown to have some role in the development of glaucoma [31-34]. Polymorphisms of glutathione S-transferases (GST) enzymes, being one of the enzymatic antioxidant systems, have been also reported to be associated with the development of glaucoma [5,35-37].

*XRCC1* is a gene that is emerging as an essential element in the repair of both damaged bases and SSBs. *XRCC1* has been shown to have a large number of SNPs, several of which are being increasingly studied in cancer epidemiology investigations and age-related diseases, in part because of their relative high frequency in the population [10,38]. A total of 37 SNPs for *XRCC1* have been identified, 14 of which code for amino acid change, and four of which have allelic frequencies of 3% or greater. Three SNPs, which have been investigated epidemiologically, were confirmed at codons Arg280His, Arg194Trp, and Arg399Gln, with allelic frequencies of 7, 13, and 27%, respectively [10].

Many SNPs in the human *XPD* gene have also been observed at >1% frequency. About 125 have been found within introns, and most of these are probably innocuous, although some may change the splicing pattern of primary *XPD* transcripts [9]. Among these SNPs, common polymorphisms have been observed at codons 312 and 751, with allelic frequencies ranging from 6% to 34% and from 9% to 3%, respectively [10]. Previous studies suggested that the Asp312Asn and Lys751Gln polymorphisms in the *XPD* gene may influence DNA repair capacity [17,22]. We therefore investigated the frequency of polymorphisms in *XPD* codon 751 (Lys-Gln) and *XRCC1* codon 399 (Arg-Trp), which are the most frequent and the most commonly studied polymorphisms of these two well-known DNA repair genes. We did not find a statistically significant association between POAG and the *XRCC1*-Arg399Gln, and *XPD* -Lys751Gln polymorphisms in this case-control study.

Although there is an apparent divergence among the results, earlier studies have reported mainly the relationship between cancers and *XRCC1*-Arg399Gln and *XPD*-Lys751Gln polymorphisms [39-42]. The association of *XRCC1* SNPs and cardiovascular disease has also received attention [10]. At this time, no studies have evaluated the possible relationship between ocular diseases and the polymorphisms of DNA repair enzymes.

There may be some explanations regarding the results, indicating no relationship between the polymorphisms of DNA repair enzymes and the risk of POAG in the current study. First, the exposure and interaction of other genes participating in DNA damage recognition, repair and cell cycle regulation may have altered the effect of *XPD* and *XRCC1* polymorphisms [43]. Second, ethnic, genetic and environmental differences in allele frequency for the investigated polymorphisms might also affect the results in genetic studies. For example, *XRCC1* gene allele frequencies for Arg399 and Gln399 polymorphisms were found as 0.60 and 0.40, respectively, in a Turkish population [44]. Another study in Turkish population reported the frequencies as 0.37 and 0.63, respectively [45]. Results of both studies are comparable to our results. On the other hand, Park et al. [46] found the frequencies as 0.79 and 0.21, respectively, in a Korean population.

Third, different levels of exposure of certain oxidative stimuli in different individuals may also have contributed to the association between the polymorphisms of the DNA repair genes and the risk of diseases, namely POAG. Fourth, DNA repair capacity among individuals is variable and it is genetically determined. Everyone has a unique combination of polymorphic traits that modify susceptibility and response to drugs, exogenous and endogenous chemical toxins, and carcinogenic exposures. Finally, glaucoma is a multifactorial disease. Possible causes of POAG include not only mutations of specific genes, but also vascular alterations, toxic effects, and mechanical injury induced by elevated IOP [5].

In conclusion, although the sample sizes of the groups of patients with POAG and healthy controls were not sufficiently large to detect any true differences between the groups, this is the first study to evaluate the possible association between the DNA repair enzyme genes and POAG development. Our results indicate that two well-known DNA-repair enzyme polymorphisms are not significantly associated with POAG development in the study population. Further studies of the precise mechanisms leading to glaucoma development are merited.
